# Polyurethane Composite Scaffolds Modified with the Mixture of Gelatin and Hydroxyapatite Characterized by Improved Calcium Deposition

**DOI:** 10.3390/polym12020410

**Published:** 2020-02-11

**Authors:** Carayon Iga, Szarlej Paweł, Łapiński Marcin, Kucińska-Lipka Justyna

**Affiliations:** 1 Department of Polymers Technology, Faculty of Chemistry, Gdansk University of Technology (GUT), Narutowicza Street 11/12, 80233 Gdansk, Poland; igaguban@pg.edu.pl (C.I.); okseren22@gmail.com (S.P.); 2 Department of Solid State Physics, Faculty of Applied Physics and Mathematics, Gdansk University of Technology (GUT), Narutowicza Street 11/12, 80233 Gdansk, Poland; marcin.lapinski@pg.edu.pl

**Keywords:** hydroxyapatite, gelatin, bone tissue, polyurethane scaffold

## Abstract

The skeleton is a crucial element of the motion system in the human body, whose main function is to support and protect the soft tissues. Furthermore, the elements of the skeleton act as a storage place for minerals and participate in the production of red blood cells. The bone tissue includes the craniomaxillofacial bones, ribs, and spine. There are abundant reports in the literature indicating that the amount of treatments related to bone fractures increases year by year. Nowadays, the regeneration of the bone tissue is performed by using autografts or allografts, but this treatment method possesses a few disadvantages. Therefore, new and promising methods of bone tissue regeneration are constantly being sought. They often include the implantation of tissue scaffolds, which exhibit proper mechanical and osteoconductive properties. In this paper, the preparation of polyurethane (PUR) scaffolds modified by gelatin as the reinforcing factor and hydroxyapatite as the bioactive agent was described. The unmodified and modified scaffolds were tested for their mechanical properties; morphological assessments using optical microscopy were also conducted, as was the ability for calcification using scanning electron microscopy (SEM) and energy-dispersive X-ray spectroscopy (EDX). Moreover, each type of scaffold was subjected to a degradation process in 5M NaOH and 2M HCl aqueous solutions. It was noticed that the best properties promoting the calcium phosphate deposition were obtained for scaffolds modified with 2% gelatin solution containing 5% of hydroxyapatite.

## 1. Introduction

Nowadays, polyurethanes (PUR) constitute the most popular group of biomaterials typically applied as a matrix of composite materials in bone tissue regeneration. They are characterized by high biocompatibility [1], bioresorption capacity [2], and the wide range of physical–chemical and mechanical properties related to their domain structure. The strong point of the PUR is the possibility to control the degradation rate by the selection of compounds for the synthesis [3]. Despite many advantages, PUR materials possess some limitations. For example, their mechanical properties significantly differ from the mechanical properties appropriate for bone tissues [4]. According to the studies of Wang et al. [5], the properties of porous PUR materials obtained by the solvent casting/particulate leaching method depends on the PUR concentration in the solution. From 15% to 25% of PUR in the solvent, the tensile and compressive modulus was in the range of 0.1–0.8 MPa and 0.05–0.35 MPa, respectively. Additionally, Da et al. [6] observed that the compressive strength of cross-linked PUR foam obtained by the lyophilization method was equal to about 0.15 MPa. In order to increase the mechanical and biological properties of PUR implants, various modificators are commonly applied. In the case of bone tissue regeneration, the most effective modifiers are hydroxyapatite (HAp) and beta tricalcium phosphate (β-TCP). They represent the class of inorganic compounds whose chemical composition and structure resembles a natural bone [7].

Hydroxyapatite is a bioactive ceramic that belongs to the calcium phosphate salts group [8]. It is mainly composed of calcium and phosphorus atoms (Ca_10_(PO_4_)_6_(OH)_2_) [9]. Hydroxyapatite is often applied as a biomaterial in bone damages (for example craniofacial bones), in joint defects, in the production of dental implants, and as a filler of scaffolds in tissue engineering [10]. Naturally, it is a major compound occurring in the human bones and teeth that is characterized by good biocompatibility as well as high osteoconductivity and osteoinductivity [11]. Despite the suitable characteristics of HAp promoting bone tissue regeneration, its low mechanical properties generate problems with using it as an independent scaffold for load-bearing bones. On the other hand, it is a desirable component for preparing modified polymeric tissue scaffolds, because it permits controlling the kinetics of polymer matrix biodegradation [12]. That is why the literature provides many examples of the HAp applications in tissue engineering as a modifier, improving the bioactivity of composite with natural or synthetic polymers [13]. 

Over the past few years, great attention has been devoted to the synthesis of nanocomposite polymeric scaffolds containing HAp as a bioactive solid signal. The presence of HAp in a composite constructs matrix may improve bone tissue formation by osteoblasts acting as a natural inorganic bone phase. Guarino et al. [14] obtained the polycaprolactone–hydroxyapatite (PCL-HAp) highly porous composites using phase inversion and particulate leaching techniques. The study considered three different contents of HAp: 13%, 20%, and 26% vol. The maximum porosity of constructs was equal to 95% and the pore size of fabricated scaffolds reached several micrometers for micropores and a few hundred micrometers for macropores. It was found that the addition of HAp slightly reduced pore size (from 131 μm for unmodified PCL constructs to 97 μm for PCL-HAp composites with 26% of HAp) and total porosity (from 95% for unmodified PCL scaffolds to 90% for PCL-HAp constructs with 26% of HAp). It was also proved that the higher fraction of a HAp bioactive solid signal improves the structure stiffness during compression (from 0.258 to 0.496 MPa). Modified scaffolds were able to ensure good cell adhesion and high viability even in the absence of growth factors.

In the paper of De Santis et al. [15], poly(ε-caprolactone)/iron-doped hydroxyapatite (PCL-FeHAp) magnetic scaffolds for bone tissue regeneration were fabricated and characterized. FeHAp nanoparticles were obtained by an acid–base neutralization conducted by heating and stirring a water suspension of FeCl_2_, FeCl_3_, and Ca(OH)_2_ at 40 °C. A Fe/Ca ratio was equal to 20% mol. and the molar ratio (Fe + Ca)/P reached 1.67. The precipitate was separated using the centrifugation technique, freeze-drying, and sieving at 150 μm. PCL granulate was dissolved in tetrahydrofuran (THF), and FeHAp nanoparticles were added to PCL/THF solution during stirring. A PCL/HAp ratio was equal to 80/20 (*w*/*w*). The obtained PCL–FeHAp nanocomposite granulate was used to fabricate porous constructs using the 3D fiber deposition process. It was confirmed that the application of FeHAp could improve the wettability and magnetic properties of polymeric constructs. In vitro assays evaluated that the bone marrow stem cells (BMCSs) growth in magnetized composite scaffolds was significantly increased (2.2-fold greater) in comparison to non-magnetized constructs. Additionally, in vivo experiments results on rabbits showed that PCL–FeHAp porous scaffolds were entirely filled with newly rebuilt bone tissue after 4 weeks. It has been shown that the control of the scaffolds’ magnetic properties can significantly improve the regeneration of bone tissue.

Moreover, Abdal-hay et al. [16] obtained the fibrous medical grade PCL–HAp composite scaffolds. The presence of HAp influenced the faster degradation of the composite constructs compared with the neat PCL scaffolds in the alkaline environment. Furthermore, it was demonstrated that the addition of HAp significantly enhanced interactions between human osteoblast cells and PCL–HAp material in comparison to unmodified PCL (higher cell viability). In the work of Kozlowska et al. [17], collagen–hydroxyapatite (Col–HAp) porous scaffolds were fabricated using the freeze-drying method. The biological properties investigations of scaffolds with the utilized mouse fibroblast cells line NIH 3T3 were performed. According to the in vitro cytotoxity assays, the cells revealed tolerance toward Col–HAp scaffolds. The absence of necrosis and the lack of abscess formation were confirmed. The paper of He et al. was related to obtain the novel material in the form of bone-like scaffolds. They were made of Col–HAp composite, which was modified with the microfibrillated cellulose (MFC). The in vitro studies demonstrated that the materials were non-cytotoxic and did not cause the hemolysis of tested cells [18]. The composite material made of alginate–hydroxyethyl cellulose–HAp was proposed by Tohamy et al. [19] as a novel material in bone tissue engineering. Performed examinations confirmed the favorable effect of HAp on the mechanical properties of produced constructs, as well as the viability and proliferation of human mesenchymal stem cells.

Additionally, the literature also provides examples of gelatin (Gel) appliance as a modifier of polymeric constructs for medical applications. Currently, it is commonly used as a component of biocomposites for bone tissue applications [20,21]. Gel is a biopolymer mostly of animal origin (pig skin, bovine hides, bovine bones [22], or fish [23]). However, due to occurring undesirable effects or unsatisfied properties of Gel derived from animals [24], different ways of production have been developed, for example by using yeast, bacteria, or plants. The structure of Gel consists of peptides and amino acids such as glycine, proline, and hydroxyproline, which provide the cell attachment to the surface of the bone tissue scaffold [25]. The basic advantages of the Gel are its solubility in aqueous medium, biocompatibility, hydrophilicity, low cost, and common availability [26,27]. Gel has also been approved as a safe product by Food and Drug Administration (FDA) [24]. Moreover, the significant impact on the mechanical properties is widely described in the literature. For example, Yin et al. [28] obtained the poly(L-lactide) (PLLA) porous constructs by using the gradual precipitation method. Then, produced scaffolds were soaked in a gelatin solution. In the case of modified scaffolds, the significant increase of mechanical properties and water uptake ability were observed. The compressive moduli of PLLA and PLLA–Gel constructs were equal to 0.57 MPa and 46.41 MPa, respectively. Furthermore, Luo et al. [29] proposed three-dimensional nanofibrous bioglass–gelatin (BG–Gel) nanocomposites’ scaffolds and noted the influence of the Gel on improvement of mechanical properties. The compressive stress of BG was equal to 9.9 kPa, while for BG/Gel, it was in the range of 27.9–34.2 kPa at 60% strain, respectively. Furthermore, Serra et.al [30] prepared scaffolds based on different formulations of chitosan (CH), Gel, and β-TCP. The compressive strength of the CH–Gel composite constructs was higher (0.12 MPa) in comparison to neat CH scaffolds (0.7 MPa). Moreover, Hossan et al. [31] obtained porous composite constructs for bone tissue engineering made of HAp and Gel. Scaffolds manufactured by mixing the mineral and organic compounds together were characterized by high thermal stability (over 300 °C) and a crystalline structure proper for a calcium phosphate polymorph with a Ca/P ratio equal 1.65. Those properties prove the utility of Gel–HAp porous constructs in the process of bone tissue.

Additionally, in the article published by Raucci et al. [32], Gel tissue scaffolds were investigated and bioactivated with inorganic solid signal (HAp) or organic compound (analog of BMP-2 peptide). Gel type B (from bovine skin) was used to fabricate porous constructs by dissolving in deionized water (5–10% wt/v) at 40 °C and processing by freeze drying in molds by 48 h. The cross-linking process was conducted by soaking obtained Gel constructs in acetone–water solution (4:1 *v*/*v*) including 1-ethyl-(3,3-dimethylaminopropyl) carbodiimide hydrochloride (EDC) (0.7% wt./v. in reference to the acetone–water solution volume). The biomineralization process was performed by immersing cross-linked Gel porous constructs in saline body fluid (SBF). HAp nuclei formation lasted 3 days, and crystalization was conducted for 4 days. Modification by organic BMP-2 was performed by covalent functionalization using microvawe-assisted solid phase peptide technology. The cross-linking process of Gel led to chemical bonding between amino and acid groups from Gel and carbodiimide bonds from EDC, which caused the decrease of chanis mobility in a hydrogel matrix. Scaffolds with 5% content of EDC showed higher swelling properties (approximately 800% for 6 h of cross-linking reaction) than constructs containing 10% of EDC (approximately 400% for 6 h of cross-linking reaction). In vitro assays confirmed that both HAp and BMP-2 modified Gel scaffolds improved the bioactivity of the obtained constructs. The first approach increased the early osteogenic differentiation of human mesenchymal cels over a short period of time, while the BMP-2 peptide improved their behavior over a longer time through the extended releasing of peptide from the scaffold’s structure.

According to the mentioned papers, a biocomposite material made of PUR and Gel as a reinforcing agent could be a promising perspective for biomedical applications. Gel is natural derivative of collagen that shows great biocompability and supporting properties. Furthermore, Gel is also used to produce controlled drug delivery systems [33], so it is good choice to obtain modifying solution for porous PUR constructs, which is made of Gel and HAp. The addition of natural ceramic materials to the scaffolds could also improve their biocompatibility and hence the chance of successful bone regeneration.

In this paper, the fabrication process of unmodified PUR scaffolds and composite scaffolds made of PUR, HAp, and reinforcing Gel was described. The materials were tested for compressive strength and strain after compression. Moreover, measurements of the size, type, and amount of pores were carried out with optical microscopy. The results of scanning electron microscopy (SEM) and energy dispersive X-ray spectroscopy (EDX) evaluated the ability of materials to calcification. Additionally, a degradation test of unmodified and Gel–HAp modified PUR scaffolds was provided in 5M KOH and 2M HCl aqueous solutions for 15 days at 37 °C.

## 2. Materials and Methods

### 2.1. Reagents

The synthesis of PUR material was carried out using: amorphous α,ω-dihydroxy (ethylene-butylene adipate) (dHEBA) macrodiol (M_w_ = 2000 Da, hydroxyl number = 54–58, acid number-max. 0.5, viscosity = 500–700 mPas at 75 °C) provided by Purinova (Bydgoszcz, Poland) (trade name POLIOS 55/20), aliphatic 1,6–hexamethylene diisocyanate (HDI) supplied by Sigma Aldrich (Poznań, Poland) (purity > 99%, density = 1.047 g/mL) and 1,4-butanodiol (BDO) as a chain extender (purity = 99%, boiling point at 230 °C), which was obtained from Purinova (Bydgoszcz, Poland). Furthermore, for the fabrication of scaffolds, dimethyl sulfoxide (DMSO) provided by Sigma Aldrich (Poznań, Poland) was used in the role of the solvent for PUR (anhydrous, purity > 99.9%). The hydroxyapatite with an average pore size diameter of < 200 nm and purity of > 97% was also obtained from Sigma Aldrich (Poznań, Poland). Gelatin was supplied by Gellwe (gelatin type B1 180).

### 2.2. Synthesis of Polyurethane 

The synthesis of PURs was described in our previous scientific papers [34,35]. Briefly, the polyurethanes were obtained by a two-step polymerization method, without a catalyst. In the first step, the prepolymer was received using the POLIOS 55/20 (77 wt %) and HDI (23 wt %). The prepolymer reaction was carried out in the glass, 4-neck reactor at 80 °C for 4 h. The next step was the chain extending process performed with BDO (the molar ratio of NCO/OH was equal to 0.9/1). After that, the reaction mixture was subjected to intensive stirring and carried into a mold with a temperature of 80 °C for 6 h. At the end, samples were transferred into a dryer at 80 °C for 24 h to complete the extending reaction.

### 2.3. Fabrication of Unmodified and Modified Scaffolds 

**Figure 1:**
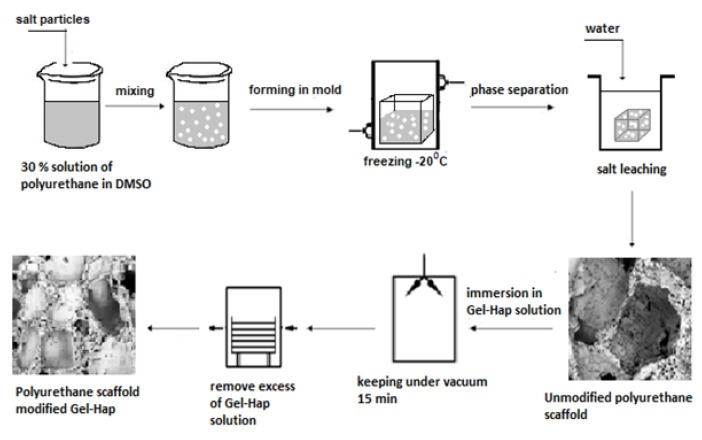
Fabrication process of scaffolds.

The PUR scaffolds were prepared using a 30% solution of polyurethane in DMSO. The process of scaffolds fabrication was based on solvent casting/particulate leaching method and is presented in Figure 1. At the first step, PUR was dissolved completely in DMSO at 85 °C. Then, salt (NaCl) with a diameter fraction in the range of 200–500 µm was added to a mixture and stirred to obtain the consistency of a paste. In the next step, paste consisting of PUR, DMSO, and NaCl was transferred into cubic molds (dimensions: 1 cm × 1 cm × 1 cm) and frozen to −20 °C. After 72 h, the obtained constructs were rinsed with deionized water to complete the removal of salt particles.

The scaffolds were modified through the soaking in the mixture of Gel and HAp. This method of modification is based on the immersion of obtained constructs in 2% Gel solution with different HAp content (1%, 3%, and 5% relative to the mass of gelatin solution). To improve the absorption of the Gel and HAp into the scaffold’s structure, the vacuum has been applied (0.1 MPa, 15 min). At the end of the modification process, the excess of Gel–HAp solution was removed, and the obtained composite scaffolds were dried at room temperature for 1 day. Symbols and descriptions of the obtained scaffolds are listed in Table 1.

### 2.4. Mechanical Properties

The compressive strength (*R_s_*) and strain after compression (*ε*) was measured using a Zwick & Roell testing machine at ambient temperature (20 °C). The measurements were carried out according to PN-EN ISO 604:2006. The cross-head speed was equal to 20 mm/min at 0.25 N cell load. Cylindrical samples (diameter = 1.4 cm, height = 1 cm) were cut out from unmodified and modified PUR scaffolds. The tests were conducted with the maximum breaking stress observation mode at ambient temperature (20 °C). Six samples of each type of scaffold were tested to obtain a mean value and standard deviation.

### 2.5. Optical Microscopy and Porosity

The morphology of obtained PUR scaffolds was observed using the Digital Microscope (Inter-Medic, Warsaw, Poland), model avp028f8 certified by FC & CE (Flood Control and Coastal Emergency) and RoHS (Restriction of Hazardous Substances). The images were observed at a magnification of ×40 and ×800 To record the microscopic images, a desktop computer with VidCap software was used. Obtained images allowed determining the size and type of pores (open or close) and additionally evaluating the interconnection of pores. To analyze microscopic images, Image J software was used. The porosity was measured using the equation presented below Equation (1):

(1) $$P=\;1-\;\left(\frac{V_{1}-V_{2}}{V_{2}-V_{3}}\right)*100\;\%$$

where *V*_1_—volume of ethanol (cm^3^); *V*_2_—volume of ethanol after immersion of scaffold (cm^3^); and *V*_3_—volume of ethanol after removal of scaffold (cm^3^).

### 2.6. Calcification Study

The calcification study was carried out according to the protocol of Golomb and Wagner. The metastable solution for calcification consisted of following components: 3.87 mM CaCl_2_, 2.32 mM K_2_HPO_4_, yielding an atomic ratio of calcium to phoshorus (Ca/P) = 1.67, and 0.05 M Tris Buffer (C_4_H_11_NO_3_) dissolved in deionized water. The preparation recipe is described thoroughly in [36]. The investigations were performed using three weighted and dried samples of each material. The area of every cylindrical sample was equal to 0.5 cm^2^ (diameter = 0.8 cm), and the thickness was 1.5 mm. Prepared samples were stored in Golomb and Wagner’s solution, and the progress of the calcification process was evaluated after 21 days.

### 2.7. Scanning Electron Microscopy and Energy Dispersive X-ray Spectroscopy

The morphology changes between unmodified and modified PUR scaffolds were studied by FEI QUANTA 250 FEG SEM microscope (Thermo Fisher Scientific, Waltham, MA, USA) at an accelerating voltage equal to 5 kV and magnification of ×700 and ×1000. The energy dispersive X-ray analyzer has been used to determine the atomic composition of scaffolds before and after calcification process. The scanning time of one survey was 200 s. Prior to the study, unmodified and modified PUR scaffolds were cut into cylindrical samples (diameter = 0.8 cm, area = 0.5 cm^2^, thickness = 1.5 mm) and covered with a conductive layer of gold by a sputter coater Quorum 150T E (Quorum Technologies, Laughton, East Sussex, UK). During the tests, tilt angle was not applied. Peaks from gold were removed from the EDX spectra (Thermo Fisher Scientific, Waltham, MA, USA).

### 2.8. Degradation Test

Studies of the short-term degradation were carried out in 5 M KOH and 2 M HCl aqueous solution. Round samples (diameter = 0.7 cm, area = 0.38 cm^2^, thickness = 0.5 cm) were cut out from unmodified and modified PUR scaffolds. Three samples taken from each type of scaffold were stored in the 1.5 mL solution of the selected medium. The degradation process was performed at 37 °C for 15 days. Before the studies, samples were dried and weighted using a Thermobalance (RADWAG MAX50/SX, Radwag Balances and Scales, Warsaw, Poland) set at 60 °C. After the degradation process, the samples were rinsed with distilled water, dried, and weighed again. The mass loss of the samples was measured by following Equation (2).

(2) $$M=\left(\frac{m_{i}-m_{0}}{m_{0}}\right)\times 100\;\%$$

where *M*—mass loss (%); *m*_0_—initial mass (g); and *m*_i_—mass after degradation process (g).

### 2.9. Statistical Analysis

The statistical analysis was performed with the use of the Origin Pro 8.5 software (OriginLab Corporation, Northampton, MA, USA). To determine the statistical differences, one-way ANOVA (*α* = 0.05), two-way ANOVA (*α* = 0.05), and a post-hoc Tukey test (*α* = 0.05) were used.

## 3. Results

### 3.1. Mechanical Properties

**Figure 2:**
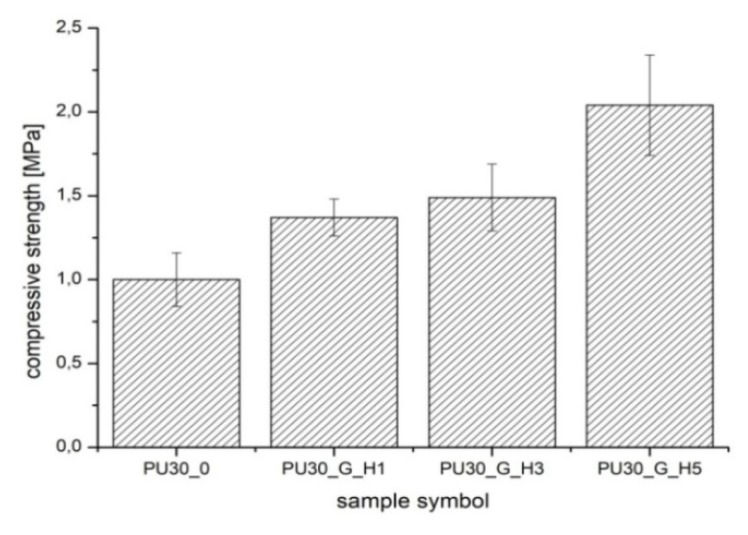
The compressive strength of PUR scaffolds.

**Figure 3:**
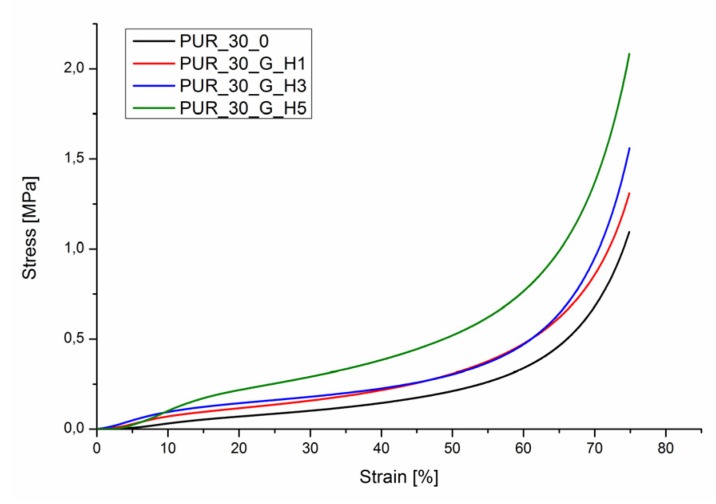
Compression stress–strain curves for PUR30_0, PUR30_G_H1, PUR30_G_H3, and PUR30_G_H5.

The unmodified scaffolds (PUR30_0) (Figure 2) were characterized by a compressive strength (*R_s_*) equal to 1.00 ± 0.16 MPa. The modification enhanced this value up to 2.04 ± 0.30 MPa for the PUR30_G_H5 containing the highest content of HAp. One-way ANOVA evaluations determined that those values are statistically different (*α* = 0.05). Stress–strain curves (Figure 3) also confirmed the highest value of compressive strength for PUR30_G_H5 samples.

**Figure 4:**
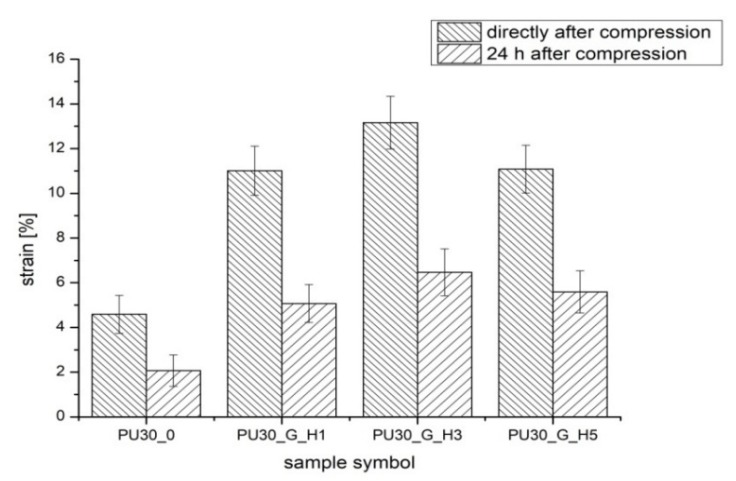
The strain directly after compression and after 24 h of PUR scaffolds.

The strain (Figure 4) directly after compression (*ε*_1_) of modified PUR scaffolds after modification significantly increased from 4.59 ± 0.85% (PUR30_0) to 11.08 ± 1.07% (PUR30_G_H5) (α = 0.05). Two-way ANOVA tests confirmed that after 24 h, the value of strain (*ε*_2_) was significantly lower in comparison to strain directly after compression for all the samples (*α* = 0.05). For instance, the strain after the compression of PUR30_G_H3 was 13.16 ± 1.18%, while after 24 h, the value significantly decreased to 6.47 ± 1.05% (*α* = 0.05).

### 3.2. Optical Microscopy and Porosity

**Figure 5:**
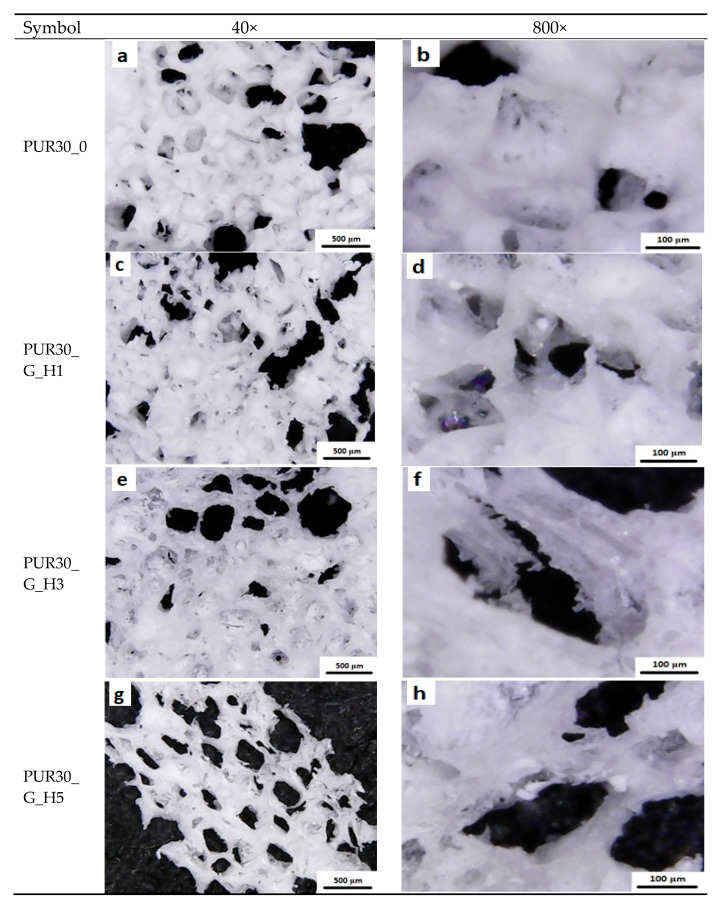
Morphology of scaffolds: (**a**,**b**) unmodified; (**c**,**d**) modified with gelatin and 1% HAp; (**e**,**f**) modified with gelatin and 3% HAp; (**g**,**h**) modified with gelatin and 5% HAp.

**Figure 6:**
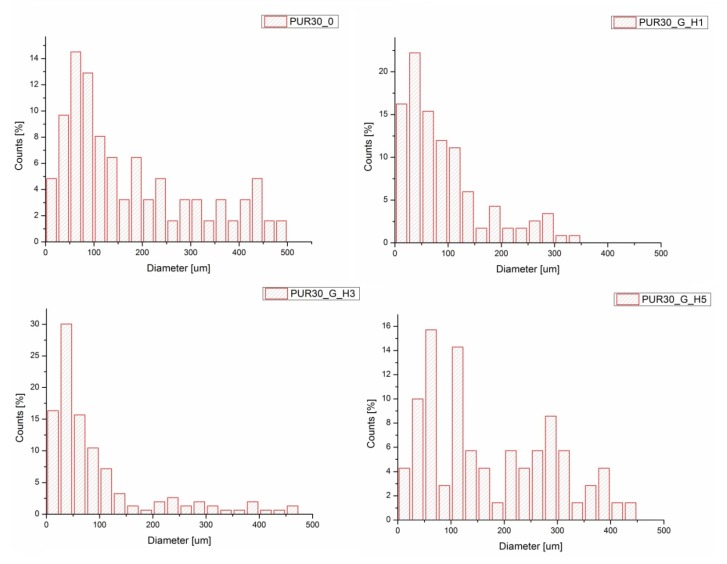
Pore size distributions for PUR30_0, PUR30_G_H1, PUR30_G_H3, and PUR30_G_H5.

It was observed that obtained unmodified and modified PUR scaffolds included both types of pores: open and close macropores with size in the range of 100–500 μm and also smaller micropores with dimensions ranging from 1 μm to 100 μm (Figure 5 and Figure 6). PUR30_G_H5 had the highest content of pores in the range of 150–500 um (47.14%), which is proper for bone tissue regeneration. PUR30_0, PUR30_G_H1, and PUR30_G_H3 contained 43.55%, 17.09%, and 16.99% of pores in the range of 150–500 µm, respectively. It was observed that modification with Gel/HAp solutions caused significant changes of the scaffold’s structure. The Gel and particles of HAp are visible on presented images. However, it is noticeable that the solidified Gel clogs the pores space to some extent and functions as a filler of porous constructs [37]. It is confirmed by the results of porosity of the scaffolds, whose value of pores decreased from 67% to 50% (Table 2). The porosity of the unmodified scaffolds (PUR30_0) was equal to 67%. The value decreased for the samples immersed in Gel–HAp solution. For PUR30_G_H5, the porosity was equal to only 50%. A one-way ANOVA test confirmed that for PUR30_0 and PUR30_G_H5, the values of porosity are statistically different (*α* = 0.05). However, for PUR30_G_H1 (60%) and PUR30_G_H3 (59.5%), the porosity parameters are not significantly different (*α* = 0.05).

### 3.3. Scanning Electron Microscopy

**Figure 7:**
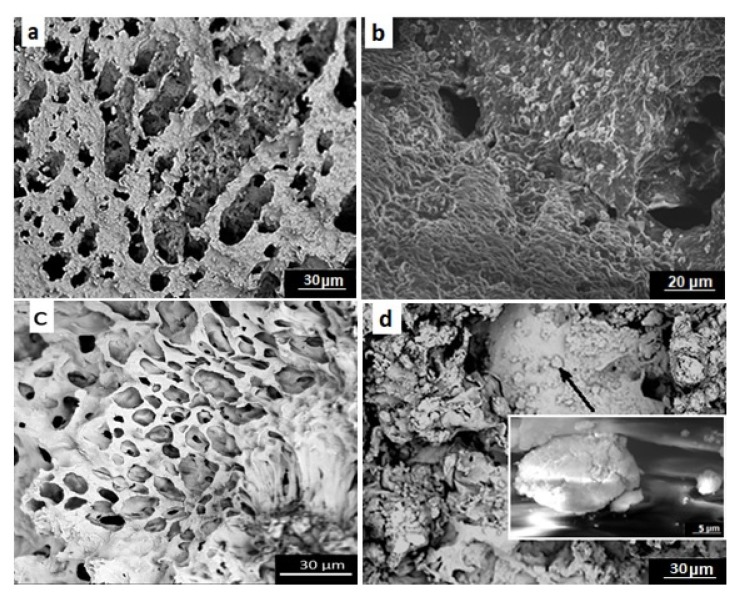
SEM images of (**a**,**b**) unmodified scaffolds before and after calcification, respectively; (**c**,**d**) scaffolds modified with gelatin and 5% HAp before and after calcification, respectively.

The SEM images of unmodified and modified scaffolds before and after calcification are presented below in Figure 7a–d. An analysis of the mentioned images revealed the adhesion of calcium compounds to both types of scaffolds (Figure 7b,d). However, a significantly greater deposition of calcium phosphate was observed in the case of PUR30_G_H5 scaffolds (Figure 7d).

### 3.4. Energy Dispersive X-ray Spectroscopy

**Figure 8:**
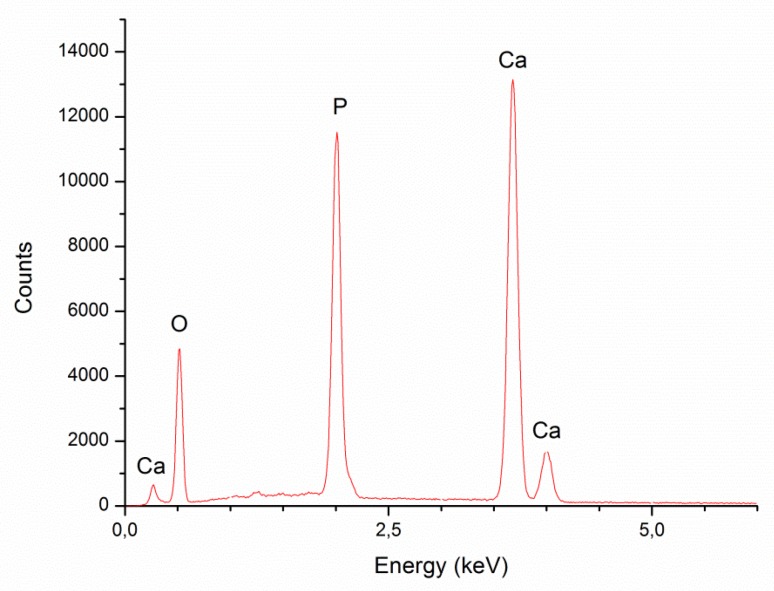
Energy-dispersive X-ray spectroscopy (EDX) spectra for HAp used for modifications of PUR scaffolds.

Figure 8 presents the EDX spectra for HAp used to obtain modified PUR scaffolds. It confirms that HAp primarily consists of calcium, phosphorus, and oxygen, which together form calcium hydroxyphosphate.

The weight and atomic composition of hydroxyapatite is shown in Table 3. The results correspond to the EDX spectra featured in Figure 8. The atomic content of oxygen, phosphorus, and calcium was equal 65.81 ± 1.78%, 13.64 ± 1.75%, and 20.55 ± 0.41%, respectively. The Ca/P ratio is ranked at 1.52 ± 0.17.

**Figure 9:**
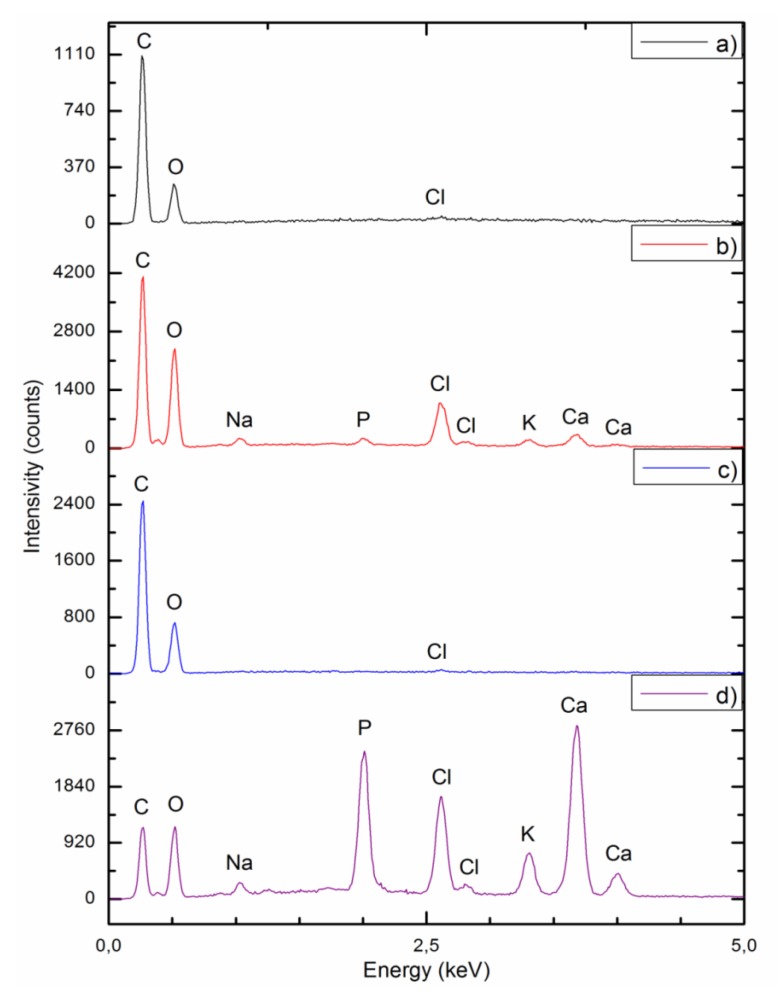
EDX spectra for (**a**,**b**) unmodified scaffolds before and after calcification, respectively; (**c**,**d**) scaffolds modified with gelatin and 5% HAp before and after calcification, respectively.

AN EDX analysis for unmodified PUR scaffolds before and after calcification and PUR composite constructs modified with gel and 5% HAp is presented in Figure 9. The research evaluated that both types of scaffolds before degradation (Figure 9a,c) consisted mainly of carbon and oxygen atoms. For both types of constructs after 21 days of calcification process (Figure 9b,d), the EDX spectra indicated the presence of carbon, oxygen, sodium, potassium, chlorine, and additionally calcium and phosphorus atoms, which proved their susceptibility to the calcification process. However, a much higher deposition of calcium phosphate was observed in the case of PUR30_G_H5 scaffolds.

The results of Ca and P deposition on the surface of PUR_30_0 and PUR_30_G_H5 after the calcification process are summarized in Table 4. For unmodified scaffolds (PUR_30_0), the atomic content of Ca and P was equal to 0.20 ± 0.05% and 0.71 ± 0.13%, whereas for modified constructs (PUR_30_G_H5), it was equal to 7.69 ± 0.83% and 13.64 ± 0.77% respectively. For PUR_30_0, the Ca/P ratio ranked at 3.63 ± 0.26, while for PUR_30_G_H5, it reached 1.78 ± 0.09. A one-way ANOVA test proved that both parameters (Ca, P content and Ca/P ratio) are statistically different (*α* = 0.05) for PUR30_0 and PUR30_G_H5.

**Figure 10:**
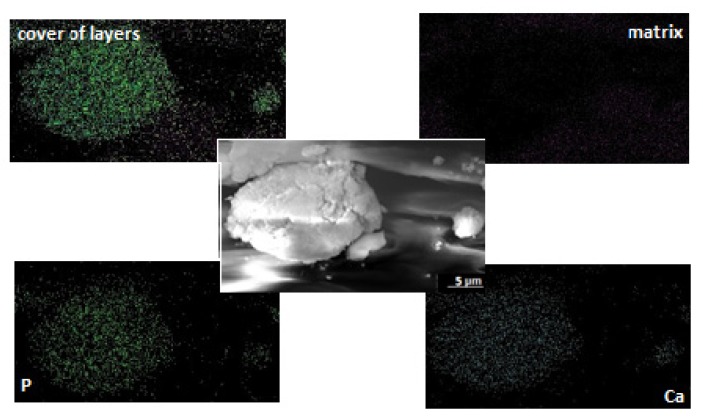
Distribution of elements in the sample of PUR30_G_H5 scaffolds.

Figure 10 presents the distribution of elements in the exemplary agglomerate of calcium phosphate deposited on the surface of PUR_G_H5 construct after calcification. Images were created using SEM in combination with EDX mapping. According to Figure 9, a homogeneous distribution of calcium and phosphorus atoms within the presented agglomerate of calcium phosphate has been demonstrated.

### 3.5. Degradation Test

**Figure 11:**
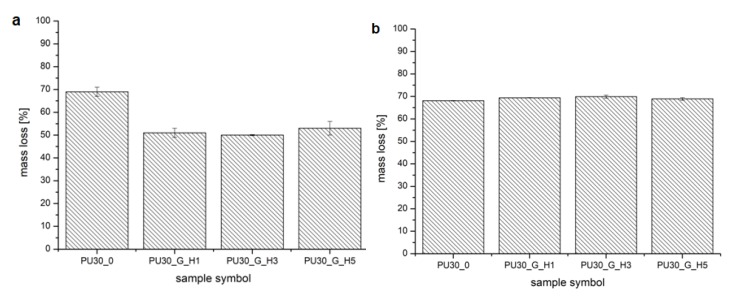
The mass loss of the scaffolds after incubation in (**a**) 5 M KOH and (**b**) 2 M HCl.

The results of mass loss of the samples are presented in the form of charts in the Figure 11a,b. The mass change of PUR_30_0 scaffolds was equal to 69.1 ± 2.3% and 68.1 ± 0.1% after a short-term degradation process in 5 M KOH and 2 M HCl, respectively. Two-way ANOVA evaluated that those mean values’ difference was not significant (*α* = 0.05). Whereas, after modification, the mass loss in 5 M KOH was significantly lower for all of the modified scaffolds in comparison to neat scaffolds (*α* = 0.05). For PUR_30_G_H1, PUR_30_G_H3, and PUR_30_G_H5, the values of mass loss were equal to 50.8 ± 1.8%, 50.3 ± 0.3%, and 52.9 ± 3.3%, respectively. In the case of the 2 M HCl medium, the results of mass loss for unmodified and modified scaffolds were very similar. As mentioned before, for PUR_30_0 constructs, the mass loss reached 68.1 ± 0.1%. In the case of PUR_30_G_H1, PUR_30_G_H3, and PUR_30_G_H5, the values were equal to 69.4 ± 0.1%, 69.9 ± 0.7%, and 68.9 ± 0.6%, respectively. A two-way ANOVA test confirmed that those values were statistically the same (*α* = 0.05).

## 4. Discussion

The bone system is a crucial part of the human body. The good condition of the skeleton allows the safe movement and proper protection of human organs. In the case of injuries, the fast reparation of damaged bones is required. One of the more modern ways of treating this kind of injuries is the application of substitutes of bones. In this paper, the unmodified and Gel/HAp modified PUR scaffolds were fabricated and tested for their mechanical properties, morphology, and behavior in various degradative solutions, which simulated the environment of the human body. Additionally, the ability of calcification was also evaluated.

The PUR scaffolds modified with gelatin and hydroxyapatite (PUR30_G_H1, PUR30_G_H3, and PUR30_G_H5) were characterized by the *R_s_* in the range of 1.37 ± 0.11–2.04 ± 0.3 MPa in correlation to the increasing amount of HAp. Values of the compressive strength of modified scaffolds were higher in comparison to the unmodified PUR30_0 scaffolds (1.00 ± 0.16 MPa). The increase of the compressive strength can be related to the reinforcing properties of Gel, what was also exhibited by Feng et al. [38], who performed the studies on the hierarchically porous calcium phosphate scaffolds (HTCP) modified with Gel. The increase of compressive strength from 5.06 MPa (neat HTCP) to 9.94 MPa (HTCP treated by 10% Gel) was observed. It is important that the compressive strength of the PUR30_G_H5 scaffold (2.04 ± 0.30 MPa) ranks in the proper range for human trabecular bone (2–12 MPa) [28,39]. In the case of the strain after the compression of scaffolds, changes in the obtained values of tested parameters were also observed. For PUR30_0, scaffolds *ε*_1_ (directly after compression) and *ε*_2_ (24 h after compression) were equal to 4.59 ± 2.3% and 2.07 ± 0.94%, respectively. Whereas, for the modified scaffolds, *ε*_1_ and *ε*_2_ reached 11.01 ± 1.73% and 5.07 ± 0.99% for PUR30_G_H1, 13.16 ± 1.18% and 6.47 ± 1.34% for PUR30_G_H3, and finally 11.08 ± 1.07% and 5.59 ± 1.96% for PUR30_G_H5, respectively.

The morphological analysis of unmodified and modified scaffolds revealed pore sizes in the range of 100–500 μm for macropores and 1 to 100 μm for micropores. The most suitable pore size for bone cells ranked in the range of 150–500 μm [40,41,42], which improved the potential utility of the obtained composite constructs in the process of bone healing. The porosity of obtained materials was changing in the range of 67%–50%. This is related to addition of gel, which significantly impacts on the decrease of porosity, according to [28,38]. It was also noticed that in the case of porous scaffolds modified through the immersion in Gel solution, Gel can act as a filler and clog the pores. However, Gel is characterized by good water solubility at 37 °C [43], so it can be a good delivery system for HAp under physiological conditions.

It is also worth to say that one of the most important parameters for materials designed for bone tissue regeneration is ability to calcify. The susceptibility of unmodified and modified materials to calcification was examined, and the results were presented in the form of SEM images and EDX spectra. Additionally, the weight and atomic content of Ca and P in PUR_30_0 and PUR_30_G_H5 after calcification are presented in Table 4. According to the SEM images (Figure 7) and EDX spectra (Figure 8 and Figure 9), the calcium phosphate deposition for both types of scaffolds was observed. EDX analysis indicated also the presence of Na, Cl, and K atoms within the scaffolds after calcification. The detection of sodium and chlorine atoms can be explained by the method of scaffolds preparation, which was based on salt leaching using NaCl, whereas potassium atoms were derived from Golomb and Wagner’s solution [36]. According to Table 4, the atomic content of Ca and P after calcification in PUR_30_0 reached 0.71 ± 0.13% and 0.20 ± 0.05%, respectively. The Ca/P ratio for unmodified scaffolds ranked in the range of 3.63 ± 0.26. In the case of PUR_30_G_H5, the significantly enhanced deposition of calcium phosphate was indicated. The atomic content reached 13.64 ± 0.77% for Ca and 7.69 ± 0.83% for P, with the Ca/P ratio in the range of 1.78 ± 0.09. It should be noted that the Ca/P ratio for PUR_30_G_H5 was very close to the proper value for hydroxyapatite occuring naturally in human bones (1.67) [44], which makes this material potentially useful for bone tissue regeneration applications.

A short-term degradation assessment indicated that for PUR_30_0, the mass loss reached 69.1 ± 2.3% and 68.1 ± 0.1% in 5 M KOH and 2 M HCl, respectively. In the case of basic hydrolysis, modified scaffolds were characterized by decreased mass loss ranking from 50.8 ± 1.8% for PUR_G_H1 to 52.9 ± 3.3% for PUR_G_H5. It was noticed that the hydroxyapatite content did not significantly affect the degradation process of the modified scaffolds in the 5 M KOH solution. In the case of acidic hydrolysis, modified constructs showed a very similar susceptibility for degradation to unmodified scaffolds. The mass change reached 69.4 ± 0.1% for PUR_G_H1 and 68.9 ± 0.6% for PUR_G_H5. Referring to these results, it was observed that for the modified scaffolds, the degradation rate was strongly dependent on the environment pH. A very similar trend was observed in our previous papers [36,45], which confirmed that poly(ester urethanes) are generally more prone to acidic hydrolysis. This information is extremely valuable from the point of view of tissue engineering, because on the basis of the type of degradation promoted by the obtained materials, it is possible to estimate how they will behave under physiological conditions.

## 5. Conclusions

The main goal of this work was to fabricate PUR scaffolds modified with mixtures of gel and HAp. These compounds have been used to reinforce the mechanical properties and improve the ability of PUR scaffolds in the calcification process, which is extremely important in the process of bone tissue treatment. Studies of mechanical properties performed for the constructs modified with the 2% Gel solution containing different amounts of HAp (1%, 3%, 5%) evaluated that the most suitable quantity of HAp equals 5%. It was observed that the presence of gelatine and hydroxyapatite caused the decrease of porosity from 67% to 50%. However, the process of Gel dissolving in the physiological conditions may increase the porosity of Gel–HAp modified constructs. Therefore, the investigations related to the calcification process (SEM images and EDX measurements) were performed only for the unmodified PUR30_0 scaffolds and PUR30_G_H5 scaffolds. It has been proven that the addition of hydroxyapatite significantly enhanced the susceptibility of modified composite scaffolds to calcification. Moreover, the Ca/P ratio of deposited calcium phosphate was very close to the value required for human bones. According to the performed tests, it can be concluded that obtained scaffolds (PUR30_G_H5) could be a suitable material for potential applications as biodegradable implants used to restore damaged bone tissue.

## Figures and Tables

**Table 1:** Symbols and descriptions of obtained scaffolds. HAp: hydroxyapatite, PUR: polyurethane.

Symbol	Description
PUR30_0	Unmodified scaffolds made of 30% solution of PUR in DMSO
PUR30_G_H1	Scaffolds modified with 2% Gel–HAp solution containing 1% of HAp
PUR30_G_H3	Scaffolds modified with 2% Gel–HAp solution containing 3% of HAp
PUR30_G_H5	Scaffolds modified with 2% Gel–HAp solution containing 5% of HAp

**Table 2:** The porosity of unmodified and modified PUR scaffolds.

Symbol	Porosity (±1%)
PUR30_0	67
PUR30_G_H1	60
PUR30_G_H3	59.5
PUR30_G_H5	50

**Table 3:** Weight and atomic composition of used hydroxyapatite.

Hydroxyapatite			
Element	Weight %	Atomic %	Ca/P (atomic)
O	45.79 ± 1.24	65.81 ± 1.78	1.52 ± 0.17
P	18.38 ± 2.37	13.64 ± 1.75	
Ca	35.83 ± 0.72	20.55 ± 0.41	

**Table 4:** Weight and atomic content of Ca and P in PUR_30_0 and PUR_30_G_H5 after calcification.

	PUR30_0			PUR30_G_H5		
Element	Weight %	Atomic %	Ca/P (atomic)	Weight %	Atomic %	Ca/P (atomic)
Ca	2.02 ± 0.37	0.71 ± 0.13	3.63 ± 0.26	26.14 ± 1.47	13.64 ± 0.77	1.78 ± 0.09
P	0.44 ± 0.11	0.20 ± 0.05		11.39 ± 1.23	7.69 ± 0.83	
